# Maternal Methyl-Group Donor Intake and Neonatal Birth Size in Singleton IVF Pregnancies

**DOI:** 10.3390/nu18111693

**Published:** 2026-05-26

**Authors:** Szilvia Bokor, Regina Felső, Ildikó Csölle, Tícia Oláh, Noémi Szabó, Róbert Herczeg, Attila Gyenesei, Reka Anna Vass, Simone Funke, Tibor Ertl, Dénes Molnár

**Affiliations:** 1 Department of Pediatrics, Medical School, University of Pécs, 7623 Pécs, Hungary; felso.regina@pte.hu (R.F.); csolle.ildiko@pte.hu (I.C.); olah.ticia@edu.pte.hu (T.O.); noemi.fogl@gmail.com (N.S.); molnar.denes@pte.hu (D.M.); 2 National Laboratory on Human Reproduction, University of Pécs, 7623 Pécs, Hungary; vass.reka@pte.hu (R.A.V.); funke.simone@pte.hu (S.F.); ertl.tibor@pte.hu (T.E.); 3 Molecular Medicine Research Group, Szentágothai Research Centre, University of Pécs, 7623 Pécs, Hungary; 4 Hungarian Centre of Genomics and Bioinformatics, Szentágothai Research Centre, University of Pécs, 7623 Pécs, Hungary; herczeg.robert@pte.hu (R.H.); gyenesei.attila@pte.hu (A.G.); 5 Cochrane Hungary, Medical School, University of Pécs, 7623 Pécs, Hungary; 6 Department of Obstetrics and Gynecology, Medical School, University of Pécs, 7623 Pécs, Hungary

**Keywords:** in vitro fertilization, methyl-group donor, folic acid, choline, newborn

## Abstract

**Background/Objectives**: Maternal intake of methyl-group donors (MGD) during pregnancy may influence fetal development, yet its role in in vitro fertilization (IVF) pregnancies remains poorly understood. The aim of the present study was to investigate maternal intake of MGDs during late pregnancy and its association with neonatal outcomes in IVF versus spontaneously (S) conceived pregnancies. **Methods**: We assessed third-trimester, daily maternal intake of MGD (folate, betaine, choline, methionine, and folic acid) using a validated food-frequency questionnaire, and maternal supplement intake using a structured questionnaire. Methyl-donor nutrient score (MDNS) was calculated based on deciles of MGD intake. Serum folic acid and vitamin B12 concentrations were measured using standardized immunochemical assay. Predefined inclusion and exclusion criteria were applied. Anthropometric data were measured from singleton newborns (weight, length, head- and waist circumference, body composition) and mothers (height, weight, body composition) after delivery. Statistical analysis was conducted using R (4.1.2v). **Results**: 265 mother–child pairs were included in the study (IVF *n* = 83). Daily dietary intake of MGDs was similar between groups, but IVF mothers reported significantly higher daily folic acid (668.7 ± 1050.9 vs. 418.8 ± 419.2 µg, *p* = 0.0034) and vitamin B12 (11.07 ± 31.58 vs. 7.95 ± 29.00 µg, *p* = 0.0078) supplementation. Serum analyses were available in a subgroup (*n* = 131, IVF *n* = 61) of mothers, showing higher postpartum folate (IVF: 10.96 ± 5.54 vs. S: 8.29 ± 4.72 µg/L, *p* = 0.0064) and vitamin B12 (IVF: 288.22 ± 113.82 vs. S: 233.70 ± 78.23 ng/L, *p* = 0.0053). Maternal daily dietary choline intakes were significantly below recommendations (IVF: 251.9 ± 98.5 mg, S: 243.8 ± 106.8 mg). Among 151 singleton neonates (IVF *n* = 57), anthropometric parameters did not differ between IVF and spontaneously conceived groups and were not associated with MDNS tertiles. **Conclusions**: Maternal MGD intake during third trimester of pregnancy was not associated with neonatal anthropometric outcomes in singleton pregnancies. Consistently low dietary choline intake highlights a potential nutritional gap warranting improved dietary guidance and supplementation strategies.

## 1. Introduction

Maternal methyl-group donor (MGD) intake during gestation is central to one-carbon metabolism [1], which plays a key role in DNA methylation processes in the developing embryo [2,3]. Consequently, altered maternal MGD availability can impair fundamental cellular functions such as embryonic growth and proliferation [4], potentially leading to altered developmental programming [3,4]. This is particularly relevant, as pregnancy is characterized by a markedly increased physiological demand for MGDs to support cellular growth and epigenetic activity in both maternal and fetal tissues [5]. Nevertheless, comprehensive studies examining the combined intake of key MGDs (such as folate, referring to naturally occurring, reduced dietary forms of vitamin B9, and folic acid, the synthetic, fully oxidized form of vitamin B9, as well as methionine, betaine, and choline) throughout pregnancy and their associations with birth outcomes remain limited [6]. Moreover, to our knowledge, no research has yet addressed this question specifically in pregnancies conceived via in vitro fertilization (IVF). This issue warrants attention, as women undergoing IVF may present distinct biological, psychological, medical, socioeconomic, and dietary characteristics that could collectively shape their nutritional status and behavior, potentially also influencing MGD intake, MGD availability, and therefore one-carbon metabolism during pregnancy [7,8,9,10,11,12].

In addition to these maternal factors, the use of IVF itself may further influence one-carbon metabolism [1,3,13,14], potentially affecting reproductive success, and long-term health-related parameters in the offspring [13,15,16,17,18,19].

Pregnancy encompasses distinct windows of susceptibility during which environmental and nutritional exposures can differentially affect fetal development and long-term health outcomes [20,21]. Nevertheless, most studies investigating maternal nutrient intake in relation to birth outcomes have primarily concentrated on the preconception period and the first trimester [3]. In contrast, dietary intake during the third trimester (despite its relevance to rapid fetal growth and tissue maturation) remains more underexplored [22]. Moreover, longitudinal analyses indicate that maternal dietary habits and supplement use evolve throughout pregnancy, with notable shifts occurring by the third trimester [22,23].

The present study aims to evaluate maternal intake of MGD during the third trimester in IVF pregnancies compared to spontaneously conceived pregnancies. Additionally, we seek to investigate the relationship between maternal combined intake of main MGDs (folate, folic acid, methionine, choline, and betaine) in the third trimester of pregnancy and the offspring’s anthropometric birth parameters.

## 2. Materials and Methods

Participants were recruited as part of the Transgenerational Effects of Assisted Reproduction (ASTRAGEN) case–control study [24], conducted at the Department of Obstetrics and Gynecology, and at the Department of Pediatrics, University of Pécs, Pécs, Hungary. The study population consisted of mother–newborn pairs, including those conceived via in vitro fertilization (*n* = 83) as well as those conceived naturally (*n* = 182).

General inclusion criteria were healthy newborns requiring no medical intervention beyond routine neonatal care, maternal age between 18 and 44 years, delivery between 36 + 0 and 42 + 0 weeks of gestation. Exclusion criteria were: pregestational diabetes mellitus, pre-eclampsia, intrauterine growth restriction (IUGR), and major fetal anomalies. Of the total cohort, a subset of participants provided informed consent for blood sampling (*n* = 131) and neonatal anthropometric assessments (*n* = 151); therefore, sample sizes varied across analyses depending on data availability. These additional analyses were restricted to women without chronic hypertension, without gestational diabetes requiring pharmacological treatment beyond diet, and with singleton pregnancies.

The study was conducted in accordance with the Declaration of Helsinki and approved by the local Ethics Committee of the University of Pécs (approval number: 8753-PTE 2023). Written informed consent was obtained from all participants.

### 2.1. Anthropometric Measurements

#### 2.1.1. Mothers

Using questionnaires, we collected information about pre-pregnancy weight, height, and socio-demographic factors, including maternal education. BMI (Body Mass Index) was calculated based on the formula body weight (kg)/body height (m) (kg/m^2^). Within 24 h after delivery, weight was determined with a digital scale with an accuracy of 0.1 kg, in light clothing, without shoes. Height was measured bare-footed with a Holtain stadiometer (Holtain Ltd., Crosswell, Crymych, UK) with an accuracy of 0.1 cm. Body composition was measured by bioelectrical impedance (Tanita Inner Scan BC-543, Tanita, Tokyo, Japan). The highest level of maternal education was classified according to the International Standard Classification of Education (ISCED) 2011 and divided into two categories: low for primary/secondary/post-secondary non-tertiary education (ISCED 0–4), and high for any tertiary education (ISCED 5–8) [25].

#### 2.1.2. Newborn

We obtained birth weight and length from the hospital clinical record. Waist and head circumferences were measured with a SECA 200 type measuring tape (seca GmbH & Co. KG, Hamburg, Germany). Body composition was measured by bioelectrical impedance (BioScan touch i8 -nano, Maltron International Ltd., Essex, England) on the morning of the first postnatal day.

### 2.2. Assessment of Methyl-Group Donor Intake

A validated food-frequency questionnaire (FFQ) was translated into Hungarian and used to assess maternal intake of dietary MGD (methionine, folate, betaine, and choline) during the third trimester of pregnancy [26]. The FFQ is a semi-quantitative, self-administered questionnaire designed to estimate habitual dietary intake over the preceding 3 months. The instrument was previously developed and validated in a European population, demonstrating acceptable validity for the assessment of dietary intake of methyl-group donors [26,27]. The questionnaire was administered at enrolment, within 24 h postpartum, and participants completed it at their own pace without a strict time limit. All questionnaires were returned within approximately a 2 h study visit window. The FFQ assessed dietary intake over the preceding three months, corresponding to the last trimester of pregnancy. To assess intake of methyl-group donors (MGD) through supplementation, information on supplement use (frequency, brand/type, dosage) during pregnancy was collected using structured questions from the Maternal Questionnaire at Enrollment (Supplementary Material S1), which also covered maternal health, obstetric history, and pregnancy-related characteristics. Supplementary intake data refer to the third trimester.

### 2.3. Calculation of Methyl-Group Donor Intake Score

Based on previous studies, folate, folic acid, betaine, choline, and methionine were considered as MGD nutrients. First, participants were divided into deciles of each MGD nutrient intake. Participants in the first decile of each nutrient received a score of 1, whereas those in the last decile received a score of 10. Scores for the other deciles were distributed as well. To create a total methyl donor nutrient score (MDNS), we summed up each nutrient score for each subject. Final scores of MDNS for each participant ranged from 5 to 50. After this, the MDNS score was divided into four categories, which created four MDNS variables: MDNS1: lowest quartile (mothers with the lowest intake of all 5 MGDs), MDNS2: second quartile for MGD intake, MDNS3: third quartile for MGD intake, MDNS4: highest quartile for MGD intake (mothers with the highest intake of all 5 MGD nutrients).

### 2.4. Laboratory Tests

Venous blood samples were collected from mothers who provided informed consent into plain vacuum tubes on the morning following delivery. The Roche Elecsys Folate III and Vitamin B12 II assays are performed using electrochemiluminescence immunoassay (ECLIA) technology on Roche cobas e 801 analytical unit (Roche Diagnostics GmbH, Mannheim, Germany). These tests are fully automated and designed for the quantitative measurement of folate and vitamin B12.

### 2.5. Statistical Methods

The statistical processing of our data was performed using the R program (R 4.1.2v, R Core Team, 2021). During the analysis, either the independent samples t-test or the Wilcoxon’s rank-sum and signed rank test was applied, depending on the normality of the data. For comparisons involving more than two groups, the Kruskal–Wallis rank-sum test was used due to non-normality confirmed by Shapiro-Wilk test. Differences in proportions were assessed using the Test of Equal proportions. Before conducting the correlation analysis, we evaluated data distribution and variable relationships through visual inspection using scatter plots and normality assessment. The associations appeared approximately linear, with no evidence of monotonic but nonlinear relationships. All analyzed variables were continuous and showed no substantial skewness or extreme outliers that would violate the assumptions of Pearson correlation. Normality was additionally assessed using the Shapiro–Wilk test. To further evaluate the robustness of the findings against potential non-normality, Spearman correlations were also calculated and yielded highly consistent results, with differences between Spearman and Pearson correlation coefficients below 0.1. Therefore, for simplicity and consistency, only Pearson correlation coefficients are presented. The significance level was defined as *p* < 0.05. When comparing anthropometric parameters across the four groups defined by the MDNS score, analyses were performed using covariate-adjusted models, with *p*-values adjusted for gestational age, sex, mode of conception (IVF or spontaneous), maternal age, and BMI variables. Statistical analyses were performed using all available data for each outcome (available-case approach); therefore, sample sizes varied across analyses depending on data availability (blood sampling and neonatal assessments). Power calculation was done in R via the pwr package (1.3.0) [28].

## 3. Results

Table 1 summarizes the clinical characteristics and laboratory parameters of the women included in the study, stratified according to the way of conception.

The mean age of women in the IVF group was significantly higher compared to the naturally conceived group. No significant differences were observed between the two groups in terms of maternal pre-pregnancy body weight, body height, BMI, gestational weight gain, body fat percentage after delivery, or maternal education level. After delivery, serum concentrations of folic acid and vitamin B12 were significantly higher in the IVF group compared to the naturally conceived group. At postpartum assessment, folic acid deficiency (serum folic acid < 4 µg/L) was present in 9.8% of IVF mothers and 20.0% of spontaneously conceiving mothers (X-squared = 1.8766, *df* = 1, *p*-value = 0.17), whereas vitamin B12 deficiency (serum B12 < 200 ng/L) was identified in 26.3% and 37.1% of the respective groups (X-squared = 1.31, *df* = 1, *p*-value = 0.25).

The daily intake of MGD from food did not differ significantly between the IVF and naturally conceived groups (Table 2).

MGD intake from supplements (specifically folic acid and vitamin B12) was significantly higher in the IVF group (Table 2). During the third trimester, 95.0% of IVF mothers reported taking daily folic acid-containing supplements, compared to only 79.6% of mothers with spontaneous conception (X-squared = 6.26, *df* = 1, *p*-value = 0.012), while daily vitamin B12 supplementation was reported by 72.8% and 66.5% of mothers in the IVF and spontaneous conception groups (X-squared = 0.362, *df* = 1, *p*-value = 0.54), respectively. On average, IVF mothers consumed 59.7% more folic acid and 39.3% more vitamin B12 per day than spontaneously conceiving mothers. Methionine, betaine, and choline were not consumed as supplements by either group. When considering total folate intake (from both food and supplements), no significant differences were observed between the groups (Table 2).

**Figure 1:**
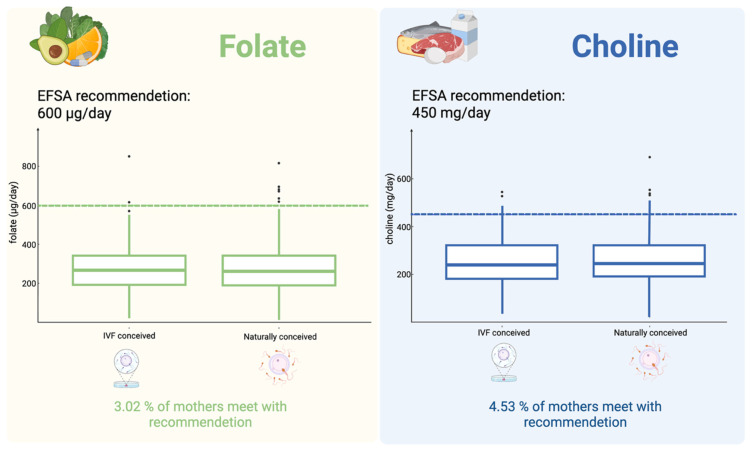
Third-trimester maternal dietary folate and choline intake in IVF and spontaneous pregnancies relative to European Food Safety Authority (EFSA) recommendations. IVF: In vitro fertilization. Units are reported according to standard nutritional reporting conventions.

The daily dietary folate intake of the investigated mothers, regardless of the mode of conception, was approximately 47% of the recommended daily intake (600 µg/day) set by the European Food Safety Authority (EFSA) (X-squared = 114.65, *df* = 1, *p*-value < 2.2 × 10^−16^) (Figure 1).

Furthermore, daily choline intake was below the EFSA-recommended level of 450 mg/day in both groups (Figure 1). On average, the investigated mothers achieved approximately 55% of the recommended daily choline intake through their diet (χ^2^ = 56.49, *df* = 1, *p* = 5.64 × 10^−14^). Only a small proportion of mothers met the recommended choline intake during the third trimester of pregnancy, including 4.9% of IVF mothers and 4.4% of spontaneously conceiving mothers (χ^2^ = 1.16 × 10^−30^, *df* = 1, *p* = 1).

From the initial cohort, a subgroup of newborns whose parents provided informed consent was included in the further analysis, comprising 57 IVF-conceived and 94 naturally conceived infants. In this subgroup, the same significant differences were observed in maternal baseline characteristics and MGD intake between the two groups: mothers in the IVF group were older and had significantly higher folic acid and vitamin B12 intake from supplements. No significant differences were found in other anthropometric parameters or in the intake of other MGD, whether from food or supplements.

Regarding the newborns, no significant differences were observed between the two groups in terms of sex ratio, gestational age, birth weight, body length, BMI, head circumference, waist circumference, or body fat percentage (Table 3).

No significant correlations were found between neonatal anthropometric parameters (body weight, body length, head and waist circumference, body fat percentage) and the maternal intake of any individual MGD (folate, folic acid, betaine, methionine, or choline) when analyzed separately (Figure S1).

To investigate the combined effect of all examined MGD, we created an MDNS score. Table 4 shows the anthropometric parameters of the newborns according to the four MDNS categories.

Newborns in the fourth MDNS category (i.e., those whose mothers had the highest intake of MGDs during the third trimester) had significantly higher birth weight compared to the other categories. After adjusting for maternal age and BMI, newborn’s gender, way of conception, and gestational age, the differences were not statistically significant.

## 4. Discussion

This study provides novel insights into maternal MGD intake during late pregnancy and its relationship with neonatal outcomes in pregnancies conceived via in vitro fertilization compared to those conceived spontaneously. Our findings demonstrate that while daily dietary intake of MGDs during the third trimester was comparable between the two groups, IVF mothers reported significantly higher supplementation with folic acid and vitamin B12, which was reflected in their elevated serum concentrations of these micronutrients after delivery. Higher intake of MGDs during the third semester of pregnancy showed no association with offspring’s anthropometric parameters.

Only a limited number of studies have assessed maternal intake of main MGD such as folate, betaine, choline, and methionine during pregnancy [6,29,30,31]. Furthermore, to our knowledge, this is the first study to specifically assess maternal dietary MGD intake in IVF pregnancies. Importantly, there was no significant difference in MGD intake between IVF and spontaneously conceived mothers, suggesting that, in terms of dietary MGD intake, IVF mothers do not exhibit higher health-conscious dietary behavior compared to spontaneously conceived mothers. While our findings regarding maternal dietary MGD intake during pregnancy generally align with previous reports from the Belgian MANOE, and ENALIA-2 cohorts [6,29,30], some differences were noted (Table S1). For example, our study found a relatively higher methionine intake compared to the MANOE studies, whereas folate intake was lower in the ENALIA-2 study. These variations may reflect the differences in dietary habits across the populations or different dietary assessment methods of the studies.

Across several studies, including our present study, a large proportion of pregnant women consistently have daily choline intakes that fall significantly below the recommended levels, highlighting a widespread nutritional gap that may warrant increased attention in perinatal care [32,33].

In this study, mothers’ daily dietary folate intake, regardless of conception mode, reached only about 47% of the EFSA’s reference value for adequate intake during pregnancy [34]. Notably, a higher proportion of IVF mothers reported folic acid supplementation during the third trimester (95.0% vs. 79.6% in the spontaneously conceiving group), which may have helped them to mitigate their relatively low dietary intake. This suggests that while IVF mothers are generally well-informed, spontaneously conceiving mothers may receive less guidance about the importance of maintaining adequate folate status during pregnancy. This may also reflect the current lack of consensus on third-trimester folic acid supplementation despite emerging supportive evidence [35,36].

Third-trimester folic acid supplementation varies considerably across countries. In our spontaneously conceiving group, 79.6% of mothers reported continued folic acid use, a higher rate compared to those observed in studies from China, Belgium, Italy, Norway, and the UK, which range between 26% and 47% [6,37,38,39], but comparable with other Belgian cohorts [40], where over 75% of pregnant women reported continued folic acid use. These differences likely reflect variations in local clinical guidelines, public health policies, and maternal counseling practices, which together shape maternal supplementation behavior during late pregnancy.

In our cohort, 9.8% of IVF mothers and 20.0% of spontaneously conceiving mothers were identified as folate deficient after delivery, which correlates with the higher folic acid intake in the IVF group. In contrast, in our study, postpartum B12 deficiency was identified in approximately 26% of mothers in both the IVF and spontaneous conception groups, despite significantly higher B12 intake from supplements in the IVF group. These results suggest that increased B12 intake may not always be translated into improved serum levels, potentially due to factors such as individual differences in absorption, metabolism, or genetic factors.

Although the majority of meta-analyses report that IVF-conceived newborns are at increased risk of preterm birth, lower birth weight, and being small for gestational age [41,42,43,44], these findings are not consistently confirmed across all studies, e.g., [45]. In our cohort, no significant differences were observed between IVF and spontaneously conceived neonates in terms of gestational age, birth weight, birth length, head circumference, waist circumference, or body fat percentage.

Epidemiological studies examining the relationship between folate and folic acid intake and birth weight have yielded inconsistent findings [46]. Although the spontaneously conceiving mothers in our study had overall lower daily intake and serum levels of folic acid compared to IVF mothers, no significant differences were observed in neonatal anthropometric outcomes between the groups. It should be noted, however, that serum folate levels may not fully capture functional folate availability. Genetic polymorphisms can impair the conversion of folic acid to its bioactive forms, potentially limiting its biological effectiveness even when serum concentrations appear adequate. The lack of this information in our study may partly explain the absence of a clear association between maternal folate status and birth anthropometric parameters.

In our study, maternal intake of individual MGD nutrients (folate, folic acid, betaine, methionine, and choline) as well as their total combined intake showed no significant association with neonatal anthropometric parameters. MGD play key roles in one-carbon metabolism, and their intake during pregnancy may influence fetal growth both directly and indirectly by modulating epigenetic programming through DNA methylation in the fetus and placenta [47,48,49]. Despite growing interest in the role of MGDs during pregnancy, few human studies have assessed their combined intake and its impact on fetal development, with most investigations examining these nutrients individually [48]. While all these nutrients are linked to fetal growth, the lack of observed associations in our study may reflect sample-specific characteristics and reflect complex interactions between nutrient intake, nutrient status, metabolism, genetic, and epigenetic factors.

### Limitations of Our Study

This study has several limitations that should be acknowledged. First, the sample size, although sufficient to detect group-level differences in supplementation habits and nutrient intake, may have limited the power to detect more subtle associations between MGD intake and neonatal outcomes. Participation in clinical assessments was consent-based, resulting in outcome-specific subsamples; thus, potential selection bias cannot be excluded. Certain maternal metabolic conditions (e.g., gestational diabetes mellitus and parity) were not systematically included as covariates, which may represent a potential source of residual confounding. Second, although a validated questionnaire was used to assess dietary intake, self-reporting may still introduce some recall bias or misreporting. Total energy intake could not be assessed due to limitations of the dietary assessment tool; thus, residual confounding by caloric intake cannot be excluded. Furthermore, the bioavailability and metabolic utilization of key MGD were not measured, which may influence the observed associations. Another limitation is the cross-sectional design, which captures dietary and supplemental intake as well as biomarker levels at a single time point (third trimester), without accounting for longitudinal changes throughout pregnancy and preconceptionally. Accordingly, dietary intake was assessed only for the preceding three months, and supplementation data were restricted to the same time period for consistency.

## 5. Conclusions

These findings contribute to the limited body of research examining the relationship between combined MGD nutrient intake in human pregnancy and offspring’s birth parameters, particularly among IVF populations. Notably, maternal third-trimester dietary folate intake was below recommendations but was compensated for by folic acid supplementation, which was more frequent in IVF than in spontaneously conceiving pregnancies, whereas choline intake was also markedly below recommended levels but not supplemented in either group. Importantly, MGD intake was not associated with adverse neonatal anthropometric outcomes in singleton pregnancies.

The results emphasize the need for enhanced education regarding both maternal dietary intake of MGDs and the appropriate use of supplements during late pregnancy, especially in spontaneously conceiving populations in Hungary. This consistently low choline intake may represent a potential nutritional gap warranting further attention through targeted dietary guidance and consideration of choline inclusion in standard prenatal supplementation.

Further research in larger and more diverse cohorts may clarify if there is a functional relevance of maternal MGD intake on fetal development and long-term offspring health.

## Figures and Tables

**Table 1:** Baseline characteristics, and measured serum folic acid and B12 vitamin levels of the investigated mothers.

	IVF Conceived (*n* = 83)	Naturally Conceived (*n* = 182)	*p*-Value
Age (years)	33.9 ± 4.9	31.0 ± 5.2	1.24 × 10^−5^ * ^t^
Body weight before pregnancy (kg)	70.7 ± 15.7	69.8 ± 16.7	0.58 ^w^
Body height (cm)	166.4 ± 7.6	164.1 ± 17.3	0.50 ^w^
BMI before pregnancy (kg/m^2^)	25.6 ± 5.6	25.4 ± 5.7	0.94 ^w^
Gestational weight gain (kg)	13.4 ± 6.7	13.2 ± 3.1	0.55 ^w^
BF percent after delivery (%)	28.4 ± 8.5	29.5 ± 7.3	0.46 ^t^
High maternal education (%)	53.1	42.0	0.13 ^p^
Serum levels after delivery	(*n* = 61)	(*n* = 70)	
Folic acid (µg/L)	10.96 ± 5.54	8.29 ± 4.72	0.0064 * ^w^
Vitamin B12 (ng/L)	288.22 ± 113.82	233.70 ± 78.23	0.0053 * ^w^

Data are shown in mean ± SD. BMI: body mass index, BF: body fat, IVF: In vitro fertilization. Baseline characteristics are presented for the total study population (IVF: *n* = 83; spontaneously conceived: *n* = 182), whereas serum folic acid and vitamin B12 concentrations are shown for the subgroup of participants who consented to blood sampling (IVF: *n* = 61; spontaneously conceived: *n* = 70). ^t^ is for Student *t*-test; ^w^ is for Wilcoxon rank sum and Signed Rank test; ^p^ is for Test of Equal proportions test from the stats R package (prop.test). * Significance level: *p* < 0.05.

**Table 2:** Daily, maternal intake of investigated MGDs.

	IVF Conceived (*n* = 83)	Naturally Conceived (*n* = 182)	*p*-Value
Daily intake of MGDs with food			
Folate (µg)	282.3 ± 137.4	279.0 ± 135.7	0.74 ^w^
Methionine(mg)	2238.2 ± 858.3	2424.6 ± 1117.4	0.19 ^w^
Choline (mg)	251.9 ± 98.5	243.8 ± 106.8	0.70 ^w^
Betaine (mg)	132.5 ± 73.5	137.6 ± 63.4	0.16 ^w^
Daily intake of MGDs with supplements			
Folate (µg)	98.96 ± 119.16	100.24 ± 150.16	0.59 ^w^
Folic acid (µg)	668.7 ± 1050.9	418.8 ± 419.2	0.0034 * ^w^
Vitamin B12 (µg)	11.07 ± 31.58	7.95 ± 29.002	0.0078 * ^w^
Daily intake of folate with supplements + food			
Folate (µg)	381.3 ± 176.1	379.2 ± 192.9	0.77 ^w^

Data are shown in mean ± SD. IVF: In vitro fertilization. MGD: methyl-group donor. Units are reported according to standard nutritional reporting conventions for each nutrient. ^w^ is for Wilcoxon rank sum and Signed Rank test. * Significance level: *p* < 0.05.

**Table 3:** Baseline characteristics of newborns.

	IVF Conceived (*n* = 57)	Naturally Conceived (*n* = 94)	*p*-Value
Boys (%)	56	64	0.56 ^p^
Gestational age (weeks)	38.7 ± 1.3	39.0 ± 1.6	0.60 ^t^
Body weight (g)	3313.8 ± 528.1	3381.9 ± 510.9	0.44 ^t^
Body length (cm)	50.1 ± 1.9	50.5 ± 2.4	0.15 ^w^
BMI (kg/m^2^)	13.2 ± 1.6	13.2 ± 1.4	0.49 ^w^
Head circumference (cm)	34.2 ± 1.9	34.6 ± 1.8	0.29 ^w^
Waist circumference (cm)	31.2 ± 3.0	31.5 ± 3.1	0.64 ^t^
BF (%)	11.3 ± 2.0	11.4 ± 2.1	0.90 ^t^

Data are shown in mean ± SD. BMI: body mass index, BF: body fat, IVF: In vitro fertilization. ^t^ is for Student *t*-test; ^w^ is for Wilcoxon rank sum and Signed Rank test; ^p^ is for Test of Equal proportions test from the stats R package (prop.test). Significance level: *p* < 0.05.

**Table 4:** Anthropometric parameters of the newborns according to the four MDNS categories.

	MDNS1	MDNS2	MDNS3	MDNS4	*p*-Value	Adjusted *p*-Value *
Gestational age (week)	38.90 ± 1.36	38.94 ± 1.57	38.08 ± 1.57	39.10 ± 1.55	0.53	
Body weight (g)	3299.19 ± 456.65	3323.24 ± 628.06	3281.19 ± 482.25	3524.08 ± 480.31	0.04	0.40
Body length (cm)	50.24 ± 1.95	49.79 ± 2.07	50.26 ± 2.55	51.00 ± 2.32	0.09	0.66
BMI (kg/m^2^)	13.04 ± 1.45	13.34 ± 1.99	12.93 ± 1.12	13.50 ± 1.26	0.25	0.45
Head circumference (cm)	34.39 ± 1.84	34.63 ± 1.76	33.77 ± 2.19	34.91 ± 1.33	0.07	0.32
Waist circumference (cm)	31.45 ± 3.47	31.19 ± 3.18	31.15 ± 2.60	31.76 ± 3.13	0.69	0.22
BF (%)	11.25 ± 2.20	10.89 ± 2.50	11.53 ± 1.85	11.57 ± 1.50	0.64	0.90

Data are shown in mean ± SD. BMI: body mass index, BF: body fat. MDNS1: lowest quartile (mothers with the lowest intake of all 5 MGDs), MDNS2: second quartile for MGD intake, MDNS3: third quartile for MGD intake, MDNS4: highest quartile for MGD intake. Significance level: *p* < 0.05. * *p* values were adjusted for maternal age and BMI, gender, way of conception, and gestational age. In all cases, the Kruskal–Wallis rank sum test was applied.

## Data Availability

Due to the sensitive nature of the data, which includes health-related information, the datasets are not publicly available. Biological samples and the associated data collected within the ASTRAGEN study are maintained as a biobank resource. Access to the data is restricted and may be granted upon reasonable request, subject to ethical approval and compliance with applicable data protection regulations.

## Supplementary Materials

The following supporting information can be downloaded at: https://www.mdpi.com/article/10.3390/nu18111693/s1, Supplementary Material S1. Selected items from the Maternal Questionnaire at Enrollment regarding dietary supplement use during pregnancy. Figure S1: Heat map of Pearson’s correlation coefficients between neonatal anthropometric parameters and maternal intake of MGDs (folate, folic acid, betaine, methionine, choline). Rows and columns represent variables included in the analysis. The color scale indicates Pearson’s correlation coefficients ranging from −1 to +1; Table S1: Maternal daily methyl-group donor intake in the third trimester of pregnancy according to different studies.

## Abbreviations

The following abbreviations are used in this manuscript:

BMI	Body Mass Index
EFSA	European Food Safety Authority
FFQ	Food-frequency questionnaire
ISCED	International Standard Classification of Education
IVF	In vitro fertilization
MDNS	Methyl-donor nutrient score
MGD	Methyl-group donors
