# Supplementary material for: Maternal Methyl-Group Donor Intake and Neonatal Birth Size in Singleton IVF Pregnancies
Source: Nutrients. 2026 May 26;18(11):1693. doi: 10.3390/nu18111693 (PMC13259345; doi:10.3390/nu18111693)
Supplement: Supplementary file 1 [file nutrients-18-01693-s001.zip › nutrients-4241498-supplementary.pdf]

Supplementary Material S1. Selected items from the Maternal Questionnaire at Enrollment regarding dietary supplement use during pregnancy

Did you take any dietary supplements or vitamin preparations during your pregnancy?
☐ Yes
☐ No

If yes, please provide the following information:
Name of dietary supplement/vitamin preparation: ____________________
Dosage: ______ tablets/day
Gestational week when supplementation was started: ______
Gestational week when supplementation was stopped: ______

Name of dietary supplement/vitamin preparation: ____________________
Dosage: ______ tablets/day
Gestational week when supplementation was started: ______
Gestational week when supplementation was stopped: ______

Name of dietary supplement/vitamin preparation: ____________________
Dosage: ______ tablets/day
Gestational week when supplementation was started: ______
Gestational week when supplementation was stopped: ______

Table S1. Maternal daily methyl-group donor intake in the third trimester of pregnancy according to different studies.

Study (Country)	Number of Subjects	Folate (µg)	Choline (mg)	Betaine (mg)	Methionine (mg)
Present study (IVF-conceived group, Hungary	n = 83	282.3± 137.4	251.9 ± 98.5	132.5 ± 73.5	2238.2 ± 858.3
Present study (Spontaneously conceived group, Hungary)	n = 182	279.0± 135.7	243.8 ± 106.8	137.6 ± 63.4	2424.6± 1117.4
MANOE study 2016 (Belgium) [31]	n = 114	268.2 ± 8.9	269.7 ± 8.0	167.4 ± 5.7	1592.1 ± 45.0
MANOE study 2017 (Belgium) [6]	n = 82	273.4 ± 102.6	273.0 ± 84.8	171.4 ± 62.5	1625.9 ± 481.8
ENALIA-2 study (Spain) [32]	n = 133	182.8 ± 368.7	271.1 ± 488.7	142.5 ± 245.9	

Data are presented as mean ± standard deviation (SD). IVF: In vitro fertilization, MANOE: Maternal Nutrition and Offspring's Epigenome, ENALIA-2: National Food Survey on adults, the elderly and pregnant women.

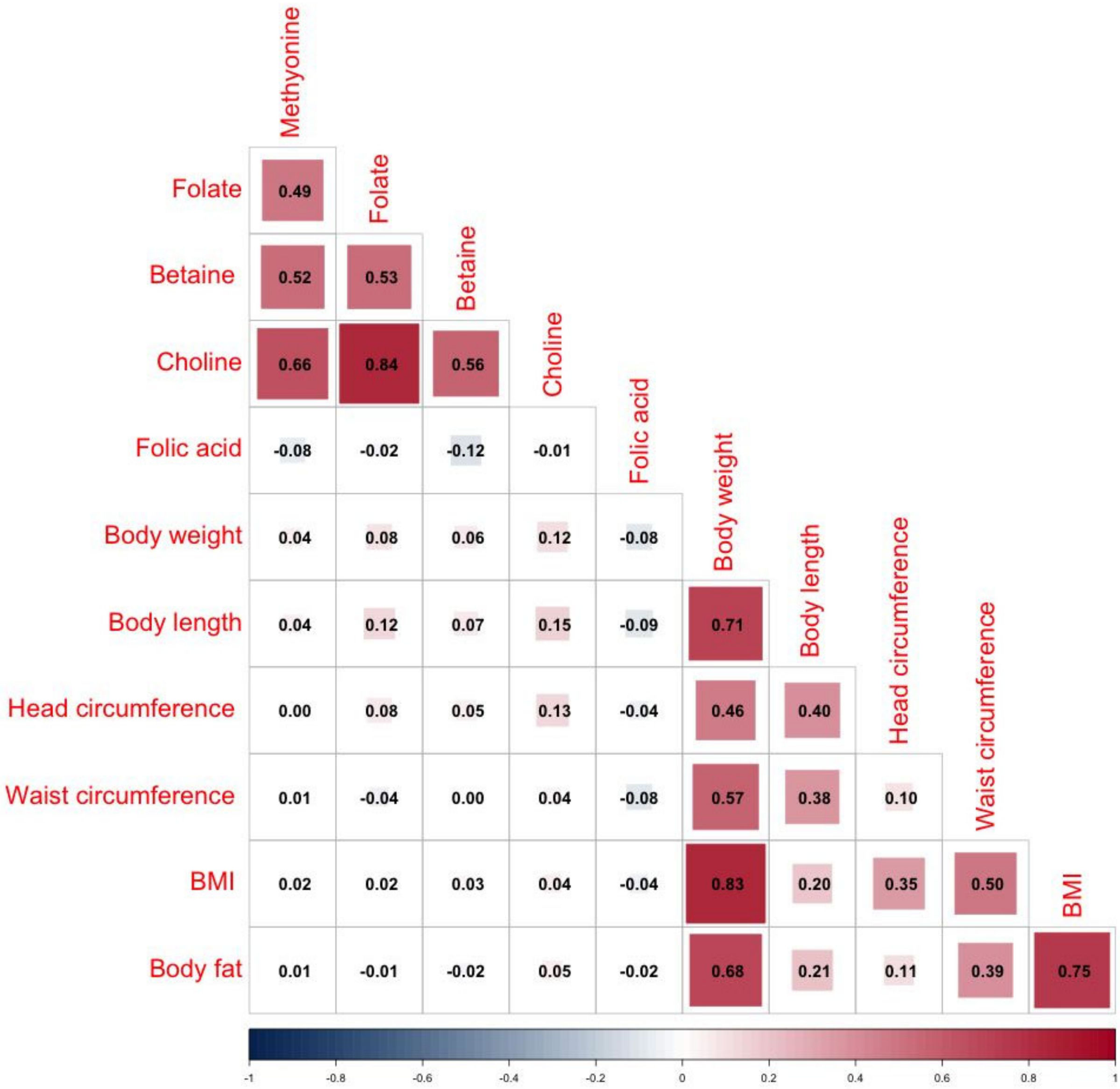

Figure S1. Heat map of Pearson's correlation coefficients between neonatal anthropometric parameters and maternal intake of MGDs (folate, folic acid, betaine, methionine, choline). Rows and columns represent variables included in the analysis. The color scale indicates Pearson's correlation coefficients ranging from −1 to +1.
